# Editorial: AI in Healthcare: From Data to Intelligence

**DOI:** 10.3389/frai.2022.909391

**Published:** 2022-05-03

**Authors:** Tim Hulsen, Milan Petkovic, Orsolya Edit Varga, Saumya Shekhar Jamuar

**Affiliations:** ^1^Department of Hospital Services and Informatics, Philips Research, Eindhoven, Netherlands; ^2^Department of Mathematics and Computer Science, Eindhoven University of Technology, Eindhoven, Netherlands; ^3^AI and Data Science Center, Philips Medical Systems, Eindhoven, Netherlands; ^4^Department of Public Health and Epidemiology, Faculty of Medicine, University of Debrecen, Debrecen, Hungary; ^5^Duke-NUS Medical School, Singapore, Singapore; ^6^KK Women's and Children's Hospital, Singapore, Singapore

**Keywords:** artificial intelligence (AI), machine learning (ML), deep learning, data science (DS), big data, healthcare, medicine

Artificial intelligence (AI), as recently defined by the European Commission, refers to systems that display intelligent behavior by analyzing their environment and taking actions—with some degree of autonomy-to achieve specific goals. AI is already being used in many industries, including healthcare, to go from “big” data to information, knowledge, and, ultimately, intelligence. Within healthcare, AI can be applied in early detection and diagnosis, treatment, outcome prediction, prognosis evaluation, and more. This Research Topic focuses on how AI is currently being used in healthcare and gives some insights into what the future might hold. It contains four articles: one on machine learning (ML) models to identify congenital heart disease (CHD), one on the predictive modeling of susceptibility to drugs in opioid patients, one on a data warehouse enabling AI on prostate cancer (PCa) data, and one that provides a qualitative and quantitative comparison of different patient modeling and simulation approaches.

Hoodbhoy et al. aim to estimate the diagnostic accuracy of ML models for detecting CHD. They collected data from 16 studies (including 1,217 participants) from the PubMed, CINAHL, Wiley Cochrane Library, and Web of Science databases. Neural networks (NN) were used in seven studies with an overall sensitivity of 90.9% and specificity was 92.7%. Other ML models included ensemble methods, deep learning (DL), and clustering techniques. In total, 69% of studies had a high risk of patient selection bias, 56% had an unclear bias on index tests, and 75% on flow and timing, while a low risk of bias was reported for the reference standard (62%). They conclude that ML models based on heart sounds acquired through a digital stethoscope have the potential to diagnose CHD with high sensitivity and specificity. Such models can facilitate earlier diagnostics in patients with suspected CHD, especially in resource limited healthcare systems. The major limitation is the heterogeneity of the diagnostic modalities used to train these models and the heterogeneity of the CHD diagnoses included between the studies.

Vunikili et al. present a collection of predictive models to identify patients at risk of opioid abuse and mortality by using their prescription histories. Using a publicly available dataset from MIMIC-III, two models were trained, Logistic Regression with L2 regularization (baseline) and Extreme Gradient Boosting (enhanced model), to classify the patients of interest into two categories based on their susceptibility to opioid abuse. The baseline model for classifying patients susceptible to opioid abuse had an F1 score of 76.64% (accuracy 77.16%) while the enhanced model has an F1 score of 94.45% (accuracy 94.35%). These models can be used as a preliminary step toward inferring the causal effect of opioid use and can help monitor prescription practices to minimize opioid abuse. They could be also efficient tools in helping uncover existing gaps and/or fraudulent practices in prescription writing.

Santaolalla et al. describe a data warehouse for the ReIMAGINE PCa project. This project aims to correct the ongoing key errors in the PCa diagnostic pathway (over-diagnosis, over-treatment, missed diagnoses, and poor risk-stratification) by combining the underlying molecular changes in prostate cancer with deep clinical phenotyping and the state-of-the-art magnetic resonance (MR) imaging. The database includes baseline clinical information, genomics, blood, urine, fresh prostate tissue samples, digital pathology, and radiomics data from two cohorts of 1,000 patients and 300 patients. Data is de-identified, stored with correlated multiparametric MRI (mpMRI) disease endotypes, and linked with long-term follow-up outcomes in an instance of the Philips Clinical Data Lake (CDL), consisting of cloud-based software. The ReIMAGINE platform includes application programming interfaces and a user interface that allows users to browse data, select cohorts, manage users and access rights, query data, and more. Connection to analytics tools such as Python allows statistical and stratification method pipelines to run profiling regression analyses.

Liventsev et al. aim to provide a solid foundation for further development and understanding of the field of interactive patient simulators. They have identified core requirements for an effective patient simulator, including transparency and explainability, difficulty, accuracy, and lack of confirmation bias in favor of currently prevalent clinical protocols. They review state-of-the-art inpatient simulators, identifying where existing solutions fall short of meeting requirements. Admitting that some of the requirements can be at odds with each other, they propose three novel simulators. HeartPole is a toy problem focused on transparency to provide as much insight as possible into the agent that interacts with the simulator. Auto-ALS is adapted from a learning aid used to train junior clinicians in Advanced Life support skills, has a focus on difficulty, and, according to the authors, could become a new benchmark in reinforcement learning. The third proposal, GraphSim, is based on the largest publically available clinical dataset and aims to maximize accuracy and eliminate confirmation bias, providing a stepping stone to improved patient outcomes in intensive care.

The articles in this Research Topic only give some examples of what AI can mean for healthcare, ranging from diagnostics (as demonstrated by papers from Hoodbhoy et al. and Santaolalla et al.) to prognostics (as demonstrated by papers from Vunikili et al. and Livenstsev et al.). There are already many opportunities to exploit AI to improve healthcare. These include predictive healthcare as part of precision medicine by suggesting preventative measures as well as the development of novel therapeutics to treat both common and rare disorders. To give just one example, healthcare providers can predict when a person is at risk of developing a chronic disease (e.g., diabetes or hypertension) and recommend preventive measures before the disease appears. However, the benefits of precision medicine are grounded in broad participation, which needs public trust, privacy protection, and a return on the value for participants. We are aware that there are a number of ethical issues and significant legal challenges that need to be addressed in introducing AI technology into everyday care. In the (near) future, AI will be poised to transform our everyday medical practice, and hopefully, we will be able to embrace and take advantage of it.

## Author Contributions

TH, MP, OV, and SJ drafted the manuscript. All authors contributed to the article and approved the submitted version.

## Conflict of Interest

TH and MP are employed by Philips. SJ is a co-founder of Global Gene Corp. The remaining author declares that the research was conducted in the absence of any commercial or financial relationships that could be construed as a potential conflict of interest.

## Publisher's Note

All claims expressed in this article are solely those of the authors and do not necessarily represent those of their affiliated organizations, or those of the publisher, the editors and the reviewers. Any product that may be evaluated in this article, or claim that may be made by its manufacturer, is not guaranteed or endorsed by the publisher.

